# Reproductive interference by male *Drosophila subobscura* on female *D. persimilis*: A laboratory experiment

**DOI:** 10.1002/ece3.2855

**Published:** 2017-03-06

**Authors:** Brenda Manzano‐Winkler, Alexander J. Hish, Emily K. Aarons, Mohamed A. F. Noor

**Affiliations:** ^1^ Biology DepartmentDuke UniversityDurhamNorth California

**Keywords:** *Drosophila persimilis*, *Drosophila subobscura*, invasive species, reproductive interference

## Abstract

While females often reject courtship attempts by heterospecific males, reproductive interference by harassment from such males can nonetheless occur, potentially reducing female fitness. Such effects may be profound following a range expansion, when males from a new species may suddenly encounter (and perhaps even become abundant relative to) females of related native species. *Drosophila subobscura* recently invaded North America and may impact native species through reproductive interference and other processes. We test for the potential for reproductive interference by *D. subobscura* males on *D. persimilis* females in the laboratory. *D. subobscura* males aggressively copulated with *D. persimilis* females, including many females that exhibit rejection behaviors. Despite females attempting to dismount the males, the heterospecific copulations are on average longer than conspecific copulations, and females exhibit some reluctance to remate with conspecific males following this harassment. Females confined with both conspecific and heterospecific males produce fewer adult progeny than those with either conspecific males only or with conspecific males and distantly related *D. simulans* males that do not engage in female harassment. Overall, our results illustrate how reproductive interference by an invasive species can have negative effects on resident natural populations.

## 1 Introduction

Reproductive interference is defined as an inter‐specific interaction associated with courtship or mating that negatively affects the fitness of at least one of the associated species (Gröning & Hochkirch, 2008). For example, males attempt to mate with both conspecific females and heterospecific females in many animal species. Females may reject the courtships of heterospecific males, but males are sometimes persistent in their mating attempts (Arnqvist & Rowe, 2005). Even when no hybrid offspring are produced, harassment by heterospecific males may reduce female fitness via energy wasted in repeated rejections of persistent males and costs or injury associated with mating (especially if forced). Some authors have suggested that patterns of species coexistence might be shaped by reproductive interference rather than the more commonly studied process of resource competition (see review in Gröning & Hochkirch, 2008).

Many animal systems exhibit evidence of reproductive interference in controlled settings (e.g., Hochkirch, Gröning, & Bucker, 2007; McLain & Pratt, 1999; Takafuji, Kuno, & Fujimoto, 1997). The bean weevil genus *Callosobruchus* has been studied extensively in this regard (see review in Kishi, 2015), and physical injury due to male genital spines may explain some of the negative fitness effect of interspecies mating on females of these species (Kyogoku & Sota, 2015). Females of many Drosophila species exhibit a detrimental “insemination reaction” resulting in abdominal swelling and sometimes infertility when fertilized by heterospecific males (e.g., Patterson, 1946). Outside of insects, cane toad and other anuran males impose fitness costs on females from other species if they do not terminate amplexus in response to cues to which conspecific males respond (Hettyey et al., 2014; Shine, 2010). In all of these cases, mating‐related interactions from heterospecific males appear to reduce the survival or fecundity of females.

Natural populations of the harassed species may undergo demographic contractions or even extinction from costs associated with interspecies harassment and mating if the aggressive male's species is very abundant (Ribeiro & Spielman, 1986). These effects may be especially pronounced in situations where the aggressive male species is very common, thus increasing interaction rates with heterospecific females (Kyogoku & Nishida, 2012; Friberg, Leimar, & Wiklund, 2013; but see Hettyey et al., 2014). One scenario where this dynamic may occur is following the invasion of a new species, though so far there is only inferential evidence to support this hypothesis. For example, persistent courtship by invasive Trinidadian guppies toward native Mexican goodeid fish may be contributing to the decline of native fish (Valero, Macias Garcia, & Magurran, 2008). Because biological invasions abruptly introduce and eventually increase the abundance of foreign species into local native communities, they provide potential natural laboratories for studying effects of reproductive interference (Remnant et al., 2014), but few studies have explored this possibility directly, and the broader ecological and evolutionary effects of reproductive interference remain poorly understood (Burdfield‐Steel & Shuker, 2011).

Here, we test for the potential for reproductive interference in a model system associated with a recent invasion. Old World native *Drosophila subobscura* was first detected in North America in the early 1980s, and by the late 1980s, became the most abundant obscura‐group Drosophila species in multiple locations along the west coast (e.g., Ayala, Serra, & Prevosti, 1989). In the laboratory, male *D. subobscura* court females of the native *D. persimilis*, often forcibly mating with them despite female resistance. No offspring are produced from this interspecies mating, yet the frequency of such matings in the laboratory is reportedly comparable to those of *D. persimilis* intra‐species matings (Wallace & Dobzhansky, 1946). In contrast, *D. persimilis* males rarely mate with *D. subobscura* females in the laboratory (Wallace & Dobzhansky, 1946), so *D. persimilis* populations bear the most likely negative consequences of potential reproductive interference between the species. *D. persimilis* has dropped in relative abundance in populations where *D. subobscura* is now common, and in some cases *D. persimilis* may have even become locally extinct (Noor, 1998). The possibility exists that these local drops may have been driven in part by reproductive interference.

The experiments presented here explore whether the potential for reproductive interference exists from invasive *D. subobscura* males on *D. persimilis* females. We verify that matings between *D. subobscura* and *D. persimilis* occur in the laboratory, we describe how the matings are aberrant relative to conspecific matings, and we show that *D. persimilis* females confined with both conspecific males and *D. subobscura* males produce fewer total progeny than those confined only with conspecific males or with conspecifics and males from distantly related species. Altogether, these results suggest the potential for reproductive interference and subsequent demographic contraction exists in this system.

## 2 Materials and Methods

### 2.1 *Drosophila* strains and culturing

*Drosophila subobscura* strains used were Seattle 6 (collected in Seattle, Washington in 2011 by Prof. Raymond Huey) and MSH 2013–12 (collected in Mount St. Helena, California, in 2013 by AJH). *D. persimilis* strains used were MSH 1993 (collected in Mount. St. Helena, California, in 1993 by MAFN) and MSH 2013–24 (collected in Mount St. Helena, California, in 2013 by AJH). *D. simulans* strain C167.4 was also used (originally collected in Nanyuki, Kenya; UC San Diego Drosophila Species Stock Center #14021‐0251.199). All flies were cultured on standard sugar/yeast/agar media and kept at 20°C with a 12:12‐hr light–dark cycle in a Percival incubator.

### 2.2 Mating behavioral observations

We used adult flies that had enclosed 5–8 days earlier for these experiments, and males were kept at low density prior to observation to reduce crowding‐mediated courtship inhibition (Noor, 1997). All mating trials were “no‐choice,” wherein a single male and female were aspirated into a plugged‐food‐containing vial and observed for one hour. Between the media and the plug, flies had roughly 50 ml of space in which to interact. All matings involved virgin male and female flies, except for 12 specified second matings that involved a virgin *D. persimilis* male and a *D. persimilis* female mated with a *D. subobscura* male several (two or more) hours earlier. The observer (AJH) recorded whether matings occurred, copulation duration, and whether the female attempted to dislodge the male during copulation using her legs and wings. Only mountings lasting at least 1 min were recorded, since virtually all the shorter ones did not achieve intromission. Copulation durations were compared using a two‐tailed Mann–Whitney *U* test, and differences in incidence of rejection behaviors were compared using a Fisher's exact test.

### 2.3 Fecundity assays

Three treatments were conducted to examine the potential of reproductive interference on fecundity. The base control treatment involved replicate vials of two *D. persimilis* males and four *D. persimilis* females, all of which were *F*_1_ progeny from a cross between the MSH 1993 and MSH 2013‐24 strains (thereby eliminating any inbreeding‐related effects). The experimental treatment had replicates of the same six flies as the base control treatment as well as eight *F*_1_ *D. subobscura* males from a cross between the MSH 2013‐12 and Seattle 6 strains. The density control treatment had replicates of the same six flies as the base control treatment as well as eight *D. simulans* C167.4 males. As implied from the label, the density control treatment had as many flies as the experimental treatment, but *D. simulans* males ignore *D. subobscura* females, so no direct reproductive interference was predicted. The subsequent test of possible effect of number of matings (conducted by EKA) had the same base control as above as well as an experimental treatment with four *D. persimilis* females and ten *D. persimilis* males.

We attempted replicates using individual or smaller numbers of *D. persimilis* females, but the food vials were likely to mold over (and thus produce few or no progeny) with anything fewer than four females. Within a replicate, all flies were confined together as adults in a food‐containing vial 5–7 days post‐eclosion. After 4–5 days of confinement, the adults were transferred to a fresh food‐containing vial for another 4–5 days and then discarded. All treatments were run in parallel with equal fly sample sizes of equal number of days of confinement, so the slight variance in number of days above cannot cause the observed differences between treatments. Progeny were allowed to develop in both vials, with a Kimwipe added as a pupation substrate. Progeny were then removed as adults from each vial and counted (by BMW). Adult progeny were counted until 28 days after setup (stopping then so as to avoid accidentally counting grandchildren), or until several consecutive days of without emergence of progeny. In total, 51 replicates of each of the three treatments were examined, though we compare the same number from each day in each statistical comparison to control for day‐to‐day environmental effects (e.g., food batch). Although we had a specific directional prediction, we conservatively used two‐sided non‐parametric Wilcoxon signed‐rank tests in *R* to assess whether the experimental treatment had fewer progeny than the controls.

## 3 Results

### 3.1 Mating behavioral observations

We observed 42 heterospecific trials of *D. subobscura* males paired with *D. persimilis* females and 24 conspecific trials of *D. persimilis* males paired with *D. persimilis* females. Among the 42 heterospecific trials, 20 resulted in copulations, and 14 of the 20 copulations elicited rejection responses by the female such as kicking the male with hind legs and pressing him with wings. These rejection responses lasted several minutes in each case. Among the 24 conspecific trials, 15 resulted in matings, and none of the 15 matings elicited the rejection responses described above. The difference in incidence of rejection behaviors differed significantly between observed heterospecific versus conspecific copulations (Fisher's exact test, *p* = .00005).

**Figure 1:**
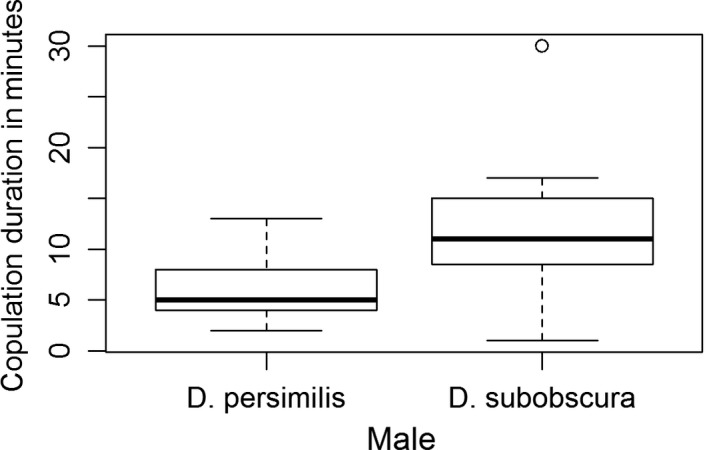
Boxplot of copulation duration of *D. persimilis* females with *D. persimilis* males and with *D. subobscura* males

Copulation duration differed significantly between the two types of matings. Despite the female rejection responses (which could have shortened copulations), copulations were on average longer in heterospecific matings (median 11 min) than conspecific matings (median 5 min; Mann–Whitney *U* test, *U *=* *43.5, *N*1 = 20, *N*2 = 14, *p* = .0008, see Figure 1).

To examine potential consequences of heterospecific matings, we chose 12 of the females who mated with *D. subobscura* and confined them with a *D. persimilis* male several (2 or more) hours later. Among these 12 females, 2 mated and 10 did not. This proportion differs significantly (Fisher's exact test, *p* = .014) from the proportion of virgin *D. persimilis* females who mated with a *D. persimilis* male (15/24: see above). Because single matings of *D. persimilis* females with *D. subobscura* males do not produce offspring but single matings of *D. persimilis* females with *D. persimilis* males obviously do produce offspring, this observation suggests that *D. persimilis* females significantly decline potentially productive (conspecific) rematings following an unproductive (heterospecific) first mating. This finding suggests a possible fecundity difference associated with reproductive interference via heterospecific first matings. We explore this hypothesis with the next set of assays.

### 3.2 Fecundity assays

**Figure 2:**
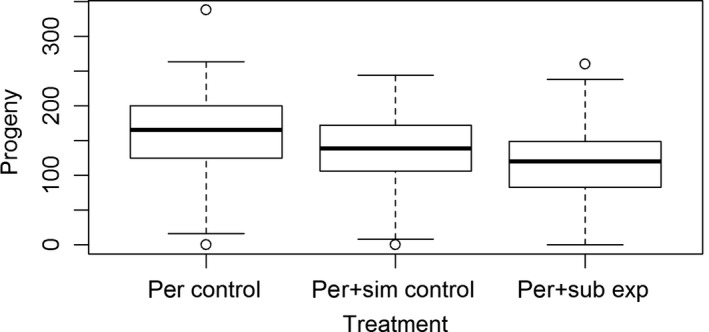
Boxplot of number of progeny collected per vial from the *D. persimilis* control (*D. persimilis* males and females), the *D. persimilis *+ *D. simulans* control (*D. persimilis* males and females with *D. simulans* males), and the *D. persimilis *+ *D. subobscura* experimental treatment (*D. persimilis* males and females with *D. subobscura* males)

We counted adult progeny from daily replicates from each of the *D. persimilis* control, the *D. persimilis *+ *D. subobscura* experimental treatment, and the *D. persimilis *+ *D. simulans* density control. The experimental treatment produced fewer offspring on average (median 120 progeny) than either the *D. persimilis* control (median 166 progeny, Wilcoxon signed‐rank test, *W* = 717, *N* = 47, *p* = .0002) or the *D. persimilis* + *D. simulans* density control (median 139 progeny, Wilcoxon signed‐rank test, *W* = 321, *N* = 43, *p* = .045, see Figure 2). The presence of *D. subobscura* males appears to have a negative effect on the number of adult progeny produced by *D. persimilis* females in captivity.

One possibility, however, is that more matings in general, rather than matings particularly with heterospecifics, may reduce reproductive output, perhaps resulting from toxic seminal fluid products (e.g., Chapman, Liddle, Kalb, Wolfner, & Partridge, 1995). We therefore repeated the control experiment (two *D. persimilis* males and four *D. persimilis* females) alongside an experimental treatment with ten *D. persimilis* males and four *D. persimilis* females. Unlike the previous experiment, the treatment with more males produced non‐significantly more offspring (*N* = 62, median offspring: 159.5) than the control (*N* = 62, median offspring: 147). Hence, the reduction in offspring numbers we document is specific to crowding with heterospecific males that court and copulate with *D. persimilis* females, consistent with reproductive interference.

## 4 Discussion

Reproductive interference by invasive species has the potential to contribute to the displacement of native species. However, few known model systems are ideal for studying ecological and evolutionary effects of reproductive interference, and several proposed cases thus far are inferential. For example, while inter‐specific matings in the groundhoppers *Tetrix ceperoi* and *T. subulata* interfere with conspecific matings in the laboratory, and although they rarely co‐occur in the same sites, it is not yet known whether the rarity of the former is due to reproductive interference (Hochkirch et al., 2007). Further, encounter rates between these species in the wild may be low as a result of microhabitat differences (Gröning, Lücke, Finger, & Hochkirch, 2007). As another example, Takakura and Fujii (2010) suggest that reproductive interference from the invasion of a cocklebur (plant) species may have caused local extinctions of the native (and endangered) *Xanthium strumarium*, but the evidence is inferential. An ideal model system for studying reproductive interference's effects would be one with collection records before and after the invader arrived and with ease of experimentation and manipulation in the laboratory and field.

Our results demonstrate that recent North American invader species *Drosophila subobscura* males actively court and often mate with North American native *D. persimilis* females, that these interspecies matings differ from conspecific matings in eliciting rejection behaviors and long copulation durations, and that confinement of the two species together reduces *D. persimilis* female fecundity in the laboratory. Altogether, these results suggest that an abundance of *D. subobscura* males may have negative fitness effects on extant *D. persimilis* populations. Since *D. persimilis* appears to be rare or absent in some natural populations that now are rich with invasive *D. subobscura*, we propose the hypothesis that reproductive interference may have contributed to the decline of *D. persimilis* in some locales. Testing this hypothesis requires field studies examining rates of interspecies mating in natural populations. Particular populations may even exhibit variation in the extent of reproductive interference, perhaps resulting from selection favoring resistance behaviors by native *D. persimilis* populations analogous to the process of reinforcement (e.g., see review in Servedio & Noor, 2003).

Until further study, we must present two caveats on the interpretations of results presented here with respect to reproductive interference. First, we cannot exclude the possibility that interactions involving the *D. subobscura* males besides those related to courtship and mating contributed to the reduced progeny numbers. For example, *D. subobscura* males may have excreted a chemical onto the media which reduced the fitness of the offspring and caused some to perish prior to adulthood. However, interspecies mating‐related interactions were observed directly, and we observed reluctance of *D. persimilis* females to remate thereafter, suggesting the potential for reproductive interference. Second, connections to natural populations in general or the potential decline of *D. persimilis* in particular are tentative. Our design was necessarily artificial since it was conducted in the laboratory and involved long periods of close confinement. It remains possible that either these species do not interact in natural populations or that they interact through resource competition or features other than reproductive interference. Now that this study has established the potential for reproductive interference in this system, the next logical step will be to test for evidence of such interactions in natural populations.

Nonetheless, this work provides a first look into the potential for reproductive interference in a model system that is highly amenable to both laboratory and field investigation. The very rapid growth of *D. subobscura* in North American populations may have had ecological impacts on endemic species, and further study will establish whether it may have impacted *D. persimilis* through mating‐related behaviors in particular, as implied from the laboratory results presented here.

## Conflict of Interest

None declared.

## Author Contributions

BMW conducted all the crosses of Drosophila strains and the experiment depicted in Figure 2. AJH collected some of the strains used from the wild and conducted the remating experiment. EKA conducted the 10‐persimilis control experiment. MAFN conceived of the project, oversaw its execution, and wrote most of the manuscript.
